# V-ATPase Proton Pumping Activity Is Required for Adult Zebrafish Appendage Regeneration

**DOI:** 10.1371/journal.pone.0092594

**Published:** 2014-03-26

**Authors:** Joana Monteiro, Rita Aires, Jörg D. Becker, António Jacinto, Ana C. Certal, Joaquín Rodríguez-León

**Affiliations:** 1 Instituto Gulbenkian de Ciência, Oeiras, Portugal; 2 Instituto de Medicina Molecular, Faculdade de Medicina de Lisboa, Lisboa, Portugal; 3 Centro de Estudos de Doenças Crónicas, Faculdade de Ciências Médicas, Lisboa, Portugal; 4 Champalimaud Foundation, Lisboa, Portugal; 5 Department de Anatomía Humana, Biología Celular y Zoología, Facultad de Medicina, Universidad de Extremadura, Badajoz, Spain; University Zürich, Switzerland

## Abstract

The activity of ion channels and transporters generates ion-specific fluxes that encode electrical and/or chemical signals with biological significance. Even though it is long known that some of those signals are crucial for regeneration, only in recent years the corresponding molecular sources started to be identified using mainly invertebrate or larval vertebrate models. We used adult zebrafish caudal fin as a model to investigate which and how ion transporters affect regeneration in an adult vertebrate model. Through the combined use of biophysical and molecular approaches, we show that V-ATPase activity contributes to a regeneration-specific H^+^ ef`flux. The onset and intensity of both V-ATPase expression and H^+^ efflux correlate with the different regeneration rate along the proximal-distal axis. Moreover, we show that V-ATPase inhibition impairs regeneration in adult vertebrate. Notably, the activity of this H^+^ pump is necessary for *aldh1a2* and *mkp3* expression, blastema cell proliferation and fin innervation. To the best of our knowledge, this is the first report on the role of V-ATPase during adult vertebrate regeneration.

## Introduction

Although humans are unable to regenerate after severe organ loss or amputation of body parts, other metazoans have such a capacity. The teleost *Danio rerio* (zebrafish) is able to regenerate several internal organs and the fins. The latter constitutes a great model to study adult vertebrate regeneration due to its easy access and non-vital function [1].

The caudal fin is composed of segmented bony rays or lepidothrichia that encircle the intra-ray mesenchyme. They are separated by inter-ray connective tissue and covered with epithelium. Blood vessels, nerves and pigment cells complete the fin [2]. Upon amputation, a new fin is produced roughly within two weeks through a process called epimorphic regeneration, including three main stages: wound healing (0–12 hours post amputation - hpa), blastema formation (12–48 hpa), and regenerative outgrowth (48 hpa to 2 weeks) [3]–[5]. Importantly, the blastema is the crucial structure for epimorphic regeneration. This heterogeneous cell population arises from dedifferentiation of mature cells [6]–[8], possibly in combination with cellular transdifferentiation and/or a resident stem cell pool, and contains the morphogenetic information required to give rise and re-pattern all the missing tissues [9]. Regeneration is regulated by the orchestrated action of several signalling pathways activated after injury, including Wnt (canonical and non-canonical), Fgf, Shh, Bmp, Activin-βA, Notch and Retinoic acid [1] [10].

Alongside classical signalling pathways, the relevance of ion channels and transporters for regeneration is becoming increasingly evident. Their coordinated activity results in the differential accumulation of ions, thus electric charge, across cells membranes. The electrical properties of all organisms arise from this charge segregation [11]. Although the generation of an endogenous wound electric current (EC) is a universal and essential response to wounding [12], the maintenance of endogenous ECs after wound closure is restricted to regenerating structures [13] [14]. In fact, it has been long known that ECs are essential to regeneration [15] [16]. In the last decade, successful efforts have begun to unveil the ionic nature of these electric cues and the molecular players that generate them. For instance, the ionic composition of the ECs at rat corneal wounds is now described and is actively regulated by specific ion transporters [17]. Moreover, cellular hyperpolarization caused by the H^+^ pump V-ATPase has proven essential for regeneration of *Xenopus* larval tail, by promoting cell proliferation and neural patterning [18]. Also, during planarian regeneration, the proton,-potassium transporter H^+^,K^+^-ATPase ensures the membrane depolarization required to specify anterior polarity in regenerating tissues and for tissue remodelling via apoptosis [19]. Other studies have identified ion transporters that generate electric signals involved in cell migration, proliferation, differentiation and apoptosis (reviewed in [20] [21]). Interestingly, all these cell behaviours are required for regeneration.

Ion transporters also generate chemical gradients that contribute to instruct specific cell behaviours [21]. For example, the increase in intracellular sodium, mediated by the voltage-gated sodium channel NaV1.2, is required for cell proliferation and tissue innervation during *Xenopus* tadpole tail regeneration [22]. In the zebrafish eye, the V-ATPase regulates retinoblast proliferation and survival, possibly through the acidification resulting from H^+^ accumulation [23]. The same H^+^ pump is essential for activation of Wnt, JNK and Notch signalling, by regulating endosomal pH [24]–[26].

Despite the increasing amount of data, a comprehensive description of the role of ion channels and transporters in regeneration is far from complete. In this study, we coupled biophysical and molecular approaches to address the ion nature and respective ion transporters involved in regeneration in an adult vertebrate (zebrafish). We show that a H^+^ outward current (efflux) is specifically set during caudal fin regeneration and that the V-ATPase, which is the main H^+^ pump in animal cells, contributes to that efflux. The onset and intensity of both V-ATPase expression and H^+^ efflux vary with the amputation plane along the proximal-distal (PD) axis in a way that correlates with the regeneration rate. Particularly, we demonstrate that inhibition of V-ATPase activity impairs regeneration and that proximal stumps have a stronger dependence on V-ATPase activity compared to distally amputated fins. We then investigated how the activity of this H^+^ pump articulates with molecular signalling pathways to affect cell behaviour and give rise to the missing tissues. We show that V-ATPase is required for *aldh1a2* and *mkp3* expression, blastema cell proliferation and normal fin innervation.

## Materials and Methods

### Ethics statement

All procedures involving animal use were approved by the Ethics Committee for Animal Welfare at Instituto Gulbenkian de Ciência (IGC), according with directives from Direção Geral de Veterinária (PORT 1005/92). All surgeries were performed under Tricaine anesthesia, and all efforts were made to minimize animal suffering.

### Animal procedures

For most experiments, we used AB wild type (WT) zebrafish (*Danio rerio*) from the IGC fish facility. The mutant line *atp6v1e1^hi577tg^* (AB) was acquired from ZIRC. All fish were raised and maintained under standard procedures [27]. Adult fish (6–9 month old) were kept at 30°C in isolated tanks with re-circulating water

Prior to manipulation, fish were anesthetized in Tricaine (Sigma-Aldrich #A5040), 1 mM for embryos/larvae and 0.6 mM for adults. Larval fin folds were amputated 2 days post-fertilization (dpf) as described [28]. Adult caudal fins were amputated with surgical razor blades along the dorsoventral axis, 1–2 ray segments before the first ray bifurcation (distal amputation) or 2 segments distal to the most posterior scale covering the fin base (proximal amputation). For amputation at both planes (proximal-distal amputation, PD), the dorsal and ventral halves of the fin were amputated at either planes; a third cut along the proximodistal axis, halfway through the dorsoventral axis, completed tissue removal. Upon manipulation, animals returned to their tanks to regenerate. For tissue collection, fins were re-amputated at several regeneration time points, 1-2 bone ray segments proximal to the first amputation.

### Scanning Ion-Selective Electrode Technique (SIET)

**Figure 1:**
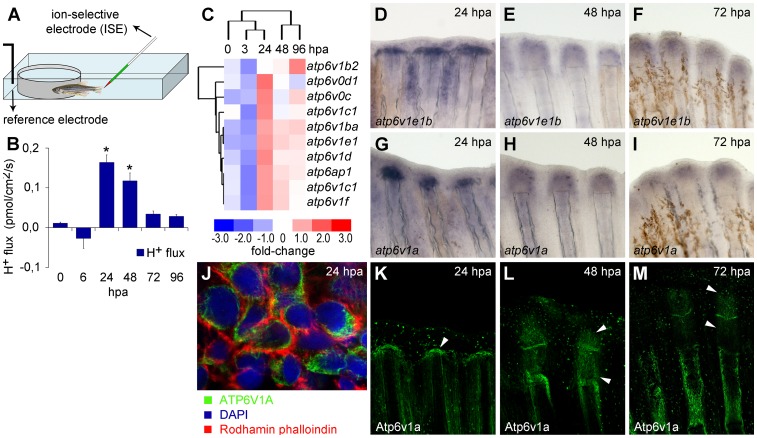
H^+^ efflux and V-ATPase upregulation accompany appendage regeneration in an adult vertebrate. (A) Recording chamber for H^+^ flux measurement using SIET, representing zebrafish emerged in recording medium. (B) H^+^ efflux established during regeneration. *statistical significant results (n = 10, p<0.05). (C) Affimetrix microarray to assess gene expression during caudal fin regeneration, compared to intact fins. (D–I) *In situ* hybridization of V-ATPase subunits *atp6v1e1b* (D–F) and *atp6v1a* (G–I), at 24 hpa (D, G), 48 hpa (E–H) and 72 hpa (F–I). (J–M) Immunohistochemical detection of Atp6v1a at 24 hpa (J, K), 48 hpa (L) and 72 hpa (M). White arrowheads point to the Atp6v1a blastema localization. (J) Detail of Atp6v1a (green) cellular localization in blastema cells, 24 hpa. hpa: hours post amputation. For each panel, n = 6, except mentioned otherwise.

SIET (Applicable Electronics LLC, USA) is a non-invasive technique that measures stable and low magnitude extracellular ion-specific fluxes in aqueous media. Ion-selective electrodes (ISE) were built, connected to the SIET system and calibrated as described in [29]–[31], with adaptations to adult zebrafish (Table S1). Fish were anesthetized in recording medium (Table S2) (adapted from [32]) and accommodated in a custom-made plate (Fig. 1A). The ISE was positioned in front of the distal end of the fin, aligned with the fin in the “X” and “Z” axes and moved between one position close to the rays (10–20 μm) and another 70 μm farther away to measure direct current (DC) voltage potential. A reference measurement was taken away from the fish. The electrical signal was recorded in ASET software (Science Wares) and transformed into ion fluxes using the Nernst equation and Fick's law, as described [33]. Fluxes were classified according to the direction: influx and efflux. We measured potassium-, sodium-, calcium-, protons- and chloride- specific fluxes. Measurements were performed at the 3^rd^, 4^th^ and 5^th^ dorsal and ventral rays. One-way ANOVA and post-hoc Tukey HSD test were used to compare H^+^ flux at all regeneration stages screened, and paired T-test for comparisons within each time point.

### Microarrays

Caudal fins were amputated at the distal plane and collected at 3, 24, 48 and 96 hpa. Intact fins (0 hpa) were also collected and used as control. Two replicates were used per time point, each consisting of three fins. Total RNA was isolated using RNeasy Mini Kit (Qiagen). Scanned arrays were analyzed first with GCOS 1.4 software to obtain Absent/Present calls and for subsequent analysis with dChip 2010 (http://www.dchip.org). The arrays were normalized to a baseline array with median CEL intensity by applying an Invariant Set Normalization Method [34]. Normalized CEL intensities of the ten arrays were used to obtain model-based gene expression indices based on a PM (Perfect Match)-only model. All genes compared were considered to be differentially expressed if the 90% lower confidence bound of the fold change between experiment and baseline was above 1.2 in both replicate datasets and if the transcript had at least one Present Call per replicate.

### Whole mount *in situ* hybridization

Gene fragments for *atp6v1e1b*, *atp6v1a*, *wnt10b*, *mkp3* and *aldh1a2* were cloned by PCR amplification from zebrafish cDNA, according to [35], using sequence-specific primers (Table S3). PCR products were ligated into pGEM-T Easy (Promega #A1360). For *atp6v1e1b*, we used pBluescript II KS+ (Stratagene #212207). Digoxigenin-labeled RNA probes were then synthesized. After collection, caudal fins were processed and used for *in situ* hybridization, as described [8]. In fins amputated at the PD plane the final precipitation reaction was stopped when the proximal regenerate was full stained.

### Whole mount immunohistochemistry

Caudal fins were fixated overnight in 0.2% PFA (for ATP6V1A) or 4% PFA (for the remaining antigens) and processed according to [8]. All methanol and acetone incubations were excluded when staining for rhodamin-phalloidin (Invitrogen R415). The following primary antibodies were used: anti-ATP6V1A (Genscript A00938), anti-Pan-cadherin (Abcam ab6528-100), anti-phospho-Histone-3 (H3P) (Millipore 06-570), anti-active-Caspase-3 (Abcam ab13847), anti-acetylated α-tubulin (Sigma T7451).

### Cell proliferation

Proliferating cells were detected by whole mount immunohistochemistry against H3P. The number of H3P-positive cells was counted in the regenerating mesenchyme and in the mesenchyme immediately below the amputation plane. We used three-dimensional projections of confocal images through the mid 20 μm of the mesenchymal depth, and pan-cadherin to exclude epithelial cells. Statistical analysis was performed using paired T-test.

### Fin fold regeneration assay

Fin folds of 15 AB WT and 15 *atp6v1e1b^hi577tg/-^* (AB) mutant larvae were amputated at 2 dpf. At 5 dpf the regenerated area was measured and compared to the area of non-amputated larvae of the same genotype using independent T-test. The two fish lines were compared separately due to genotype- specific morphometric differences. Phenotypic defects were also investigated by visual inspection.

### Pharmacological and morpholino knockdown

Different morpholinos (Gene Tools, LLC) were used to block translation of V-ATPase subunit *v1e1b*. Fluorescein-tagged morpholinos (fluo-MO) and corresponding mismatch controls (cfluo-MO) were used at 1 mM. Vivo-morpholino (vivo-MO) and control vivo-MO (cVivo-MO) were delivered at 0.5 mM. Their sequences were as follows (5′ to 3′): fluo-MO-1 TCGGCATCGCTGAGCGCCA, cfluo-MO-1 TCcGCATgGCTGAcCGCgA, fluo-MO-2 TGCAGATCCTGCTCCTGCTGCTTTA, cfluo-MO-2 TGgAcATCgTGCTgCTcCTGCTTTA, vivo-MO TCGGCATCGCTGAGCGCCA, cVivo-MO CCTCTTACCTCAGTTACAATTTTATA. Fins were amputated at either the proximal or distal level. Each *atp6v1e1b*-specific morpholino was injected into one half of the caudal fin at 2 or 16 hpa, and the other half received the corresponding control. The same number of fish received the inhibitory molecule at the dorsal and ventral part. Fluo-MO experiments were performed on AB WT fish, and vivo-MO was delivered to *atp6v1e1b^hi577aTg/+^* (AB) fish (heterozygous fish carrying a recessive mutation for *atp6v1e1b*). For fluo-MOs, injection was immediately followed by electroporation of the whole fin. Both procedures were performed as described [36]. The areas of regenerating tissue after MO and control MO delivery were compared with a paired T-test. Results were plotted as the difference (%) in treated regenerate area compared to the control, calculated using the formula: (MO area x 100/control MO area) -100 = % MO area relative to the control.

Concanamycin A (concA, Sigma C9705) was dissolved in 100% DMSO and diluted in standard Danieau medium to working concentrations of 100 and 500 nM; the corresponding control solutions were 0.1% and 0.5% DMSO respectively, both prepared in Danieau medium. ConcA and control were injected every 12 h between 6–42 hpa. The experimental design and procedure was as described for vivo-MO.

### Imaging

For *in situ* hybridization and *in vivo* protocols, images were obtained with a Leica Z6APO stereomicroscope equipped with a Leica DFC320 color camera. Immunostainings were imaged on a Zeiss LSM 710 confocal microscope. The ImageJ software was used to analyze the Z stacks and to measure regenerate area and blastema length. All statistical analyses were done on SPSS 16.0 software.

## Results

### Proton efflux accompany blastema formation

The individual contribution of K^+^, Na^+^, H^+^, Ca^2+^ and Cl^−^ - specific fluxes to the ECs during adult zebrafish fin regeneration was investigated using SIET (Fig. 1B, File S1). From the five ion-species tested, H^+^ was the only one with a dynamic pattern in stages specific to regeneration events (later than wound healing). Before amputation (0 hpa) and during wound healing (6 hpa) fins maintained a small H^+^ efflux close to the background noise (p>0.05, independent T-test). However, by 24 hpa, when the wound had healed and a blastema was forming, an outward current was established. This efflux was 14-fold higher than the efflux detected in intact fins (p<0.05, one-way ANOVA), and remained at high intensity until the end of blastema formation (48 hpa). From 72 hpa on, it decreased towards levels closer to the uninjured tissue (Fig. 1B). SIET measurements show for the first time that H^+^ current is specifically set during adult vertebrate appendage regeneration, suggesting that some mechanism of H^+^ extrusion is activated in cells during regeneration.

### V-ATPase is specifically upregulated during adult caudal fin regeneration

To find candidate H^+^ transporters that could be associated to regeneration and to the detected H^+^ efflux, we took advantage of an Affymetrix microarray previously done in our laboratory to assess gene expression during regeneration after distal amputation. Upon a specific analysis for proton transporters' expression, we focused on the V-ATPase, one of the main H^+^ transporters in animal cells. In fact, all V-ATPase subunits present in the microarray were upregulated between 24 and 96 hpa (Fig. 1C). This expression pattern matches that of H^+^ efflux, suggesting that the regeneration specific H^+^ extrusion could be associated to the increased V-ATPase expression.

The regeneration-associated expression of this H^+^ pump was confirmed by *in situ* hybridization for different V-ATPase subunits. Neither *atp6v1a* nor *atp6v1e1b* were detected in intact fins (Figures A and B in File S2). In contrast, 24 h after distal amputation, both *atp6v1a* and *atp6v1e1b* were expressed in the blastema-forming region and in scattered cells below the amputation plane (Fig. 1D, G and Figure C in File S2). By 48 hpa, their expression domain was expanded, accompanying the growth of the blastema (Fig. 1E, H). At 72 hpa both transcripts were faint but could still be observed in the distal regenerating region (Fig. 1F, I).

For V-ATPase detection at the protein level, we targeted the cytoplasmic subunit Atp6v1a. In the intact fin, Atp6v1a was mainly restricted to the epidermis, in a scattered pattern (Figures D-F in File S2). On the contrary, by 24 hpa, this subunit was strongly upregulated in the blastema (Fig. 1J, K) and was present all over the blastema until48 hpa (Fig. 1L). At that time, Atp6v1a also became evident in the regions where ray segment joints were forming. At 72 hpa it was also present in the areas of lepidotrichia regenerative outgrowth and in the intra-ray mesenchyme (Fig. 1M). Atp6v1a did not co-localize with rhodamin- phalloidin, which labels cortical F-actin, but stained immediately below (Fig. 1J). Considering that Atp6v1a is part of the cytoplasmic domain of the H+ pump, our results did not exclude a possible plasma membrane location of the whole pump.

Overall, these three different approaches are in agreement with a specific upregulation of V-ATPase during regeneration. Both V-ATPase expression and H^+^ efflux intensity seem stronger during blastema formation and decrease thereafter, suggesting a causal relation.

### V-ATPase inhibition affects regeneration

**Figure 2:**
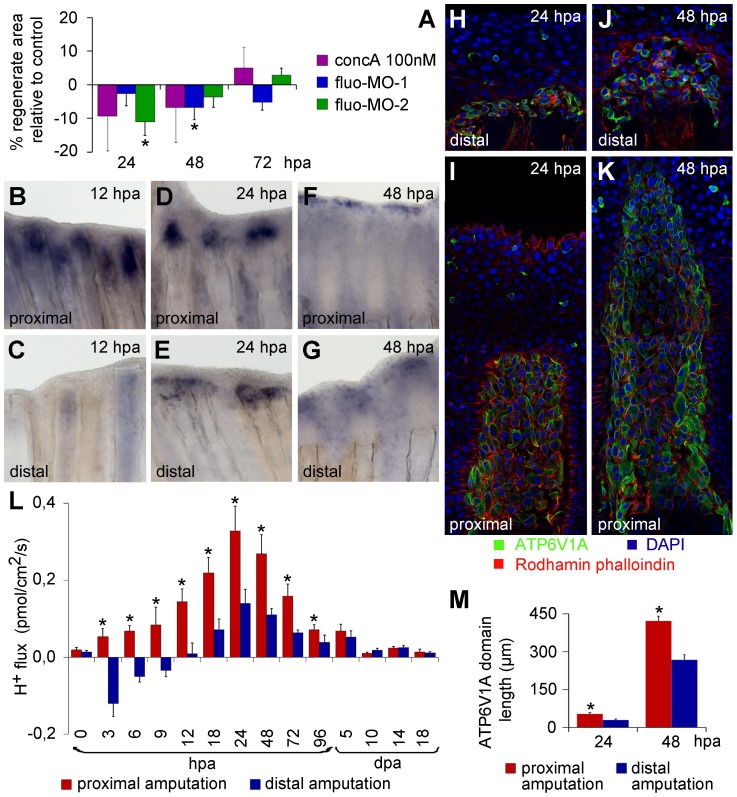
V-ATPase inhibition affects regeneration and, similar to H^+^ efflux, has a different expression pattern depending on the amputation plane along the PD axis. (A) Inhibition of the V-ATPase using concanamycinA (concA) and fluorescein- tagged morpholinos (fluo-MO-1 and 2). (B–M) Experiments performed after proximo-distal amputation. (B–G) *In situ* hybridization for *atp6v1e1b* at 12 h (B, C), 24 h (D, E) and 48 h (F, G) after proximal (B, D, F) and distal amputation (C, E, G). (H–K) Immunohistochemical detection of Atp6v1a at 24 h (H, I) and 48 h (J, K) after proximal (I, K) and distal amputation (H, J). (L) H^+^ efflux pattern during regeneration after proximal and distal amputation (n = 8–10). (M) Length of Atp6v1a expression domain in proximal and distal stumps, 24 and 48 hpa. *statistical significant results (n = 3, p<0.05). hpa: hours post amputation. For each panel, n = 6, except mentioned otherwise.

To assess the functional significance of the V-ATPase during fin regeneration, we used concA to specifically inhibit the pump's activity. Translation of a*tp6v1e1b*, a V-ATPase subunit essential for the pump activity, was also blocked using gene specific fluo-MOs. Half regenerating fin was treated with one inhibitor whereas the other half received the corresponding control (File S3). All V-ATPase inhibitors decreased the regenerate area for at least 48 h compared to the corresponding control, despite high phenotypic variability (Fig. 2A). This suggests a role for this H^+^ pump in the regenerative process. In fact, V-ATPase inhibition seemed to affect regeneration rate more that the regenerative ability itself, since, notwithstanding the reduced area, regeneration still progressed in a delayed fashion.

### V-ATPase affects H^+^ efflux expression and correlates with the position-dependent regeneration rate

To investigate if V-ATPase was related to regeneration rate, we took advantage of the fact that proximal stumps have higher regeneration rate than distal ones [37] [38], and compared V-ATPase expression after proximal versus distal (PD) amputation. In proximal wounds, *atp6v1e1b* was first visible around 12 hpa in the first ray segment below the amputation plane and, to a smaller extent, in the interray (Fig. 2B). Expression at the distal stump only became evident at 24 hpa, as described (Fig. 1F, Fig. 2C, E). By that time *atp6v1e1b* domain in proximal stumps was much stronger and wider (Fig. 2D). By 48 hpa the differences between proximal and distal *atp6v1e1* expression had faded (Fig. 2F, G). Accordingly, at the protein level, Atp6v1a in proximal stumps was present in almost twice the length than in the regions amputated distally by 24 hpa (proximal:distal length ratio mean ± s.e.m  = 1.84±0.17) (Fig. 2H, I, M). At 48 hpa, the protein domain was still 1.57±0.08 fold longer in proximal wounds (Fig. 2J, K, M). From the above, V-ATPase seems to have a dynamic expression according to the level of amputation: in the proximal, fast regenerating regions, the pump expression starts earlier and the expression domain is larger than in the slower regenerating distal wounds.

We have previously suggested V-ATPase as a mediator of the regeneration-specific H^+^ efflux. If so, the dynamic expression of V-ATPase along the PD axis should be accompanied by a concordant H^+^ efflux pattern. Thus, we measured H^+^ flux throughout regeneration after PD amputation, using SIET (Fig. 2L). The efflux peaked always at 24 hpa. However, in proximal stumps H^+^ efflux started earlier (3 hpa instead of 12 hpa) and was significantly higher than in distal regions for each time-point (p<0.05, paired T-test). This agrees with the different onset and magnitude of V-ATPase expression at proximal and distal stumps, reinforcing the correlation between H^+^ efflux and V-ATPase and extending such correlation to the position-dependent regeneration rate.

**Figure 3:**
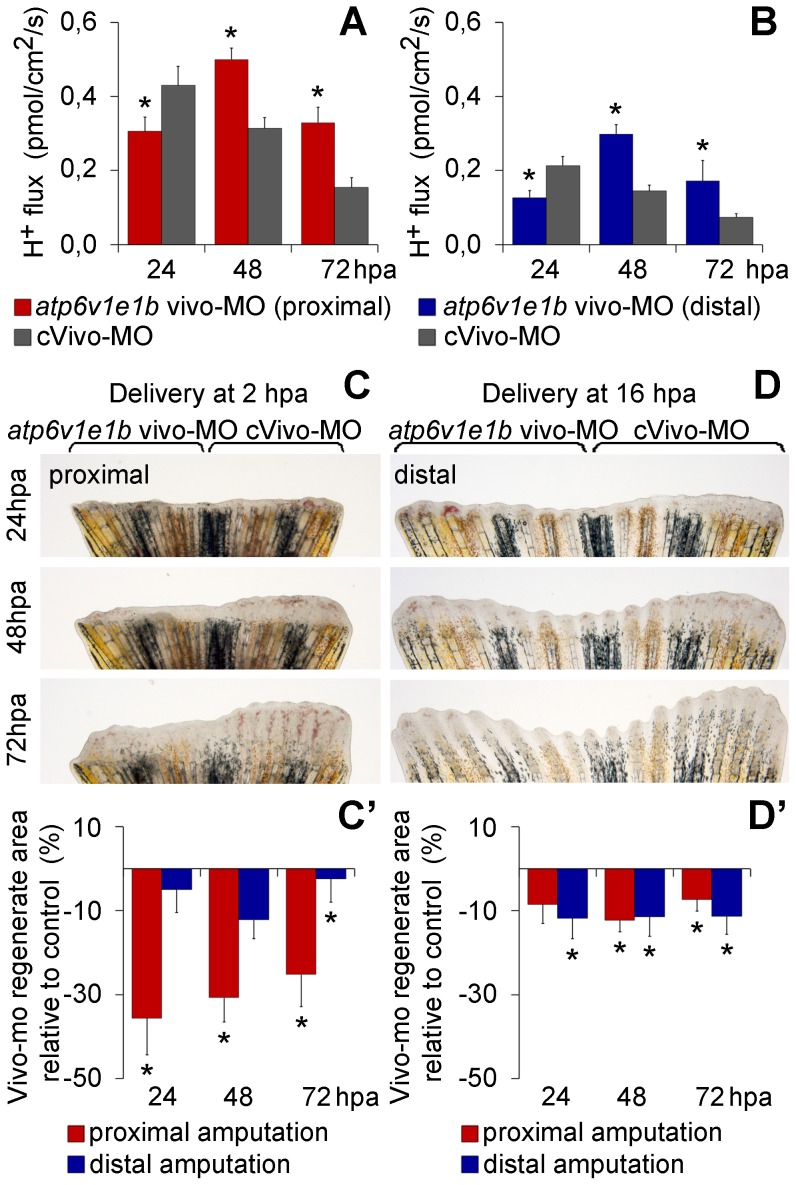
Vivo-morpholino mediated *atp6v1e1b* knockdown impairs regeneration and decreases H^+^ flux. (A, B) H^+^ flux during regeneration after vivo-MO mediated *atp6v1e1b* knockdown, 2 h after proximal (A) and distal (B) amputation. *statistical significant results (p<0.05). (C, D) Caudal fin regeneration after *atp6v1e1b* vivo-MO delivery to *atp6v1e1b^hi577aTg/+^* (AB) fish, 2 h after proximal amputation (C) and 16 h after distal amputation (D). (C’, D’) Percentage of regenerate area in *atp6v1e1b* knocked down regions compared to the control vivo-MO (cVivo-MO). Vivo-MOs were delivered 2 h (C’) or 16 h (D’) after both proximal and distal amputation. hpa: hours post amputation. For each panel, n = 8.

To deepen the relation between V-ATPase activity and H^+^ efflux, we analysed the effect of V-ATPase inhibition on H^+^ flux. For that, we used *atp6v1e1b^hi577aTg/+^* (AB) fish to perform an *atp6v1e1b* knockdown using vivo-MO as described in materials and methods, at 2 h after proximal and distal amputation. Then, H^+^ fluxes were measured at main regeneration stages using SIET (Fig. 3A, B). Aside from the higher efflux intensity proximally, distal and proximal wounds had a similar flux pattern, as follows: in the controls, H^+^ efflux intensity was maximal at 24 hpa and then decreased gradually, as previously detected (Fig. 2L). After *atp6v1e1b* knockdown, H^+^ efflux at 24 hpa decreased significantly (p<0.05, paired T-test), confirming the causal relation between V-ATPase activity and H^+^ efflux. Surprisingly, by 48 hpa the efflux pattern reverted compared to the control: as the latter began its descendent path, the former increased significantly (p<0.01, paired T-test) to a level that resembled the control efflux at 24 hpa for the same amputation plane. Similarly, at 72 hpa the H^+^ efflux intensity was higher than in the control (p<0.01, paired T-test), and was similar to the control efflux at 48 hpa, as if there was a 24 h delay on flux intensity due to the transient vivo-MO effect. Thus, the level of flux seems to be controlled by V-ATPase activity and relevant for position- dependent regeneration.

### V-ATPase is required during regeneration and is dependent on the amputation plane

To further investigate the role of V-ATPase and its association with regeneration rate, we performed vivo-MO mediated *atp6v1e1b* knockdown in *atp6v1e1b^hi577aTg/+^* (AB) fish, 2 or 16 h after proximal and distal amputation. In all experimental sets, *atp6v1e1b* knockdown decreased the regenerate area compared to the control (Fig. 3C, C’, D, D’). However, proximal regenerates were more affected by vivo-MO delivery at 2 hpa (Fig. 3C, C’) whereas distal stumps exhibited a more significant area reduction when *atp6v1e1b* was inhibited at 16 hpa (Fig. 3D, D’). Importantly, this correlates with the different onset of both V-ATPase expression and H^+^ efflux, and again it links to regeneration rate. Besides, the overall decrease in the regenerate area was steeper proximally than at distal stumps (24 hpa, mean±s.e.m. = 35.6±8.7% and 11.7±4.9%, respectively, for delivery at 2 and 16 hpa), suggesting that higher regeneration rate have a stronger dependence on V-ATPase activity.

### V-ATPase is not required for larval fin fold regeneration

**Figure 4:**
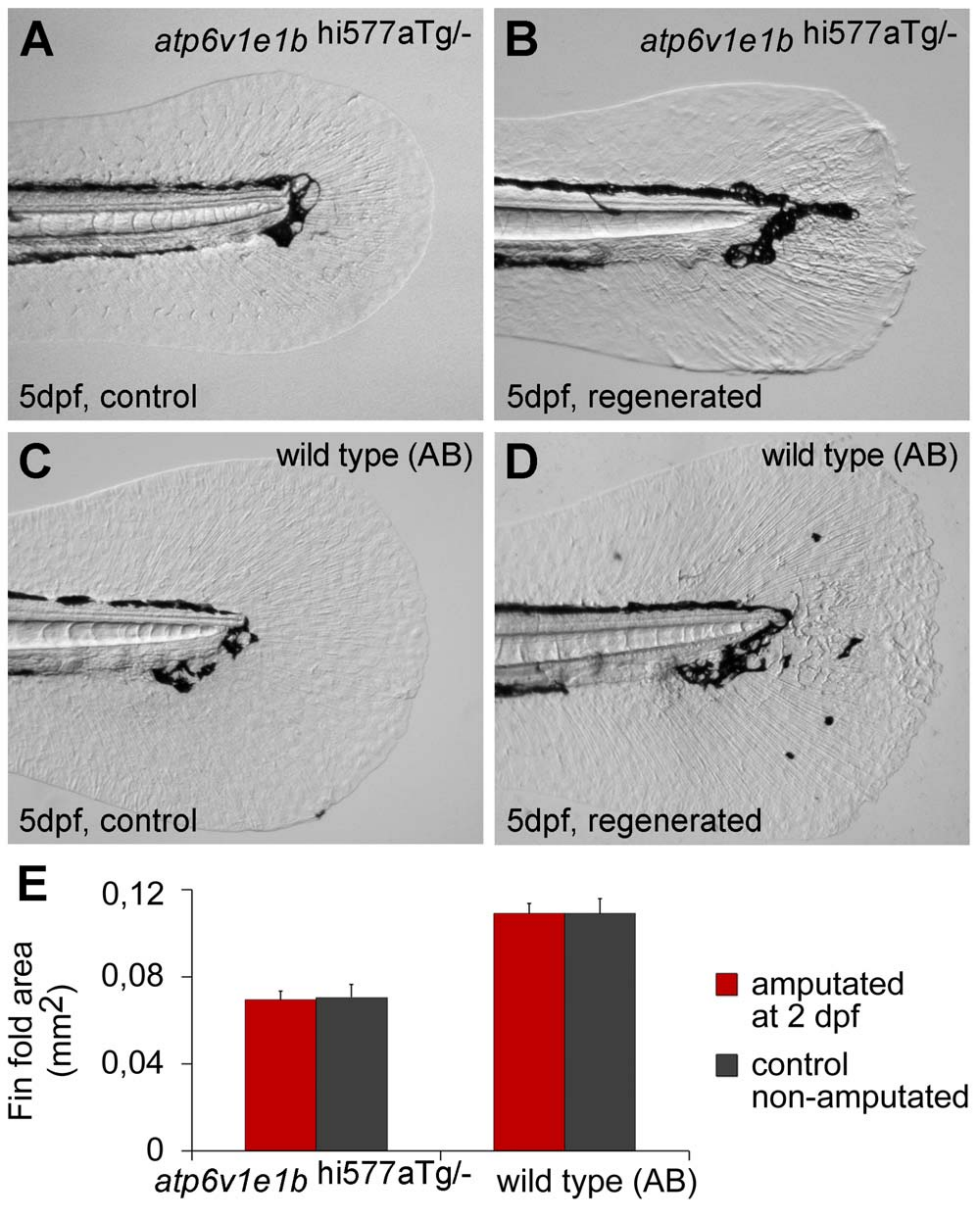
V-ATPase is not required for larval fin fold regeneration. (A, B) Fin fold of 5 dpf *atp6v1e1^hi577aTg/-^* mutant larvae, intact (A) or regenerated after amputation at 2 dpf (B). (C, D) Fin fold of 5 dpf AB wild type larvae, intact (C) or regenerated after amputation at 2 dpf (D). (E) Area of intact and regenerated fin fold by 5 dpf for either genotypes (p>0.05). dpa: days post amputation. For each panel, n = 15.

The knockout of one V-ATPase subunit becomes lethal around 6 dpf [23], long enough to study the effects of the gene absence in the larval fin fold regeneration. In fact, many evidences suggest that regeneration of adult and larval caudal appendage follow similar mechanisms [5] [28]. Given that, we amputated the fin fold of *atp6v1e1^hi577aTg/-^* mutants and AB WT fish at 2 dpf and the regenerated area was compared with non-amputated fins of the same genotype 3 days later. Intact mutants were underdeveloped and had a smaller fin fold than the wild type (Fig. 4A, C, E). After amputation, there were no additional phenotypic differences between the two fish lines (Fig. 4B, D), and the regenerated fin fold area was similar to the control, for either genotypes (Fig. 4E). These results show a normal larval regeneration process in the absence of V-ATPase activity, contrary to what was observed in the adult caudal fin. Hence, regeneration of the adult and larval caudal appendages do not seem to operate through the same mechanisms.

### Blastema cell proliferation is reduced upon *atp6v1e1b* knockdown

**Figure 5:**
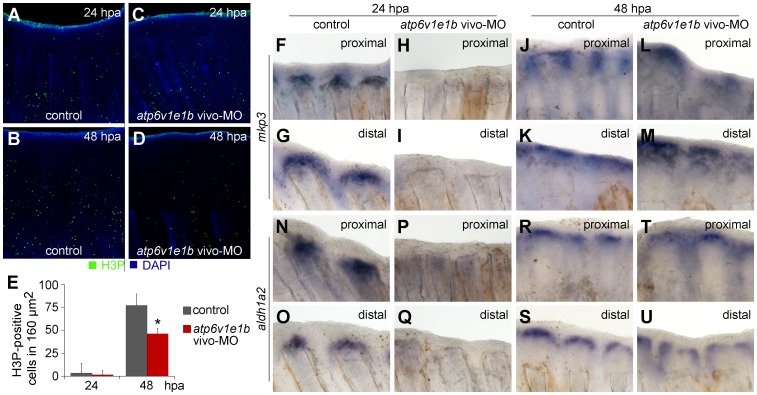
V-ATPase is required for blastema cell proliferation and expression of *mkp3* and *aldh1a2*. (A–D) Immunohistochemical detection of proliferating cells (H3P-positive) 24 h (A, C) and 48 h (B, D) after proximal amputation. (A, B) control fin; (C, D) V-ATPase knocked down fin. (E) Quantification of H3P-positive cells in the blastema. *statistical significant results (p<0.05). (F–U) *In situ* hybridization for *mkp3* (F-M) and *aldh1a2* (N-U), after proximal amputation (F, H, J, L; N, P, R, T) and distal amputation (G, I, K, M; O, Q, S, U): (F–G; N–O) control fin, 24 hpa; (H–I; P–Q) V-ATPase knocked down fin, 24 hpa; (J–K; R–S) control fin, 48 hpa; (L–M; T–U) V-ATPase knocked down fin, 48 hpa. Control fin: caudal fin from AB wild type fish, non-treated. V-ATPase knockdown procedure: *atp6v1e1b* vivo-MO delivered to the caudal fin of *atp6v1e1b^hi577aTg/+^* (AB) fish, 2 hpa. hpa: hours post amputation. For each panel, n = 3.

Reduced regeneration upon V-ATPase inhibition could be due to increased cell death or decreased cell proliferation. To investigate that, we knocked down *atp6v1e1b* in the caudal fin of *atp6v1e1b^hi577aTg/+^* (AB) fish by delivering vivo-MO at 2 hpa. Fins were collected at different regeneration timepoints and immunostained for active-Caspase-3 and Phospho-Histone-3 (H3P), apoptotic and proliferative markers respectively. Apoptosis was similar in both control and treated fish, suggesting that reduced regeneration is not due to increased apoptosis (File S4). As for proliferation, 24 h after proximal amputation, most H3P-positive cells in control fins were found in the intra-ray mesenchyme 1–2 ray segments below the proximal amputation plane, with only occasional cells stained above the amputation plane (Fig. 5A); whereas at 48 hpa, many proliferating cells had accumulated in the mature blastema (Fig. 5B). Likewise, *atp6v1e1b* knockdown didn't affect the number or location of proliferating cells by 24 hpa (Fig. 5C). However, at 48 hpa there were significantly less blastema cells positive for H3P than in the control (less 40.1%±11.58) (Fig. 5D, E). Similar results were found in distal regenerates (File S5). These results show that V-ATPase is required for normal cell proliferation in the blastema.

### V-ATPase is required for normal expression of *aldh1a2* and *mkp3*

Cell proliferation during blastema formation is controlled by several signalling pathways, including Fgf, Wnt/β-catenin and Retinoic acid (RA). We assessed the link between V-ATPase and these signalling pathways by comparing the expression of *mkp3*, *wnt10a* and *aldh1a2* during proximal and distal regeneration in control fins (AB WT fish) and in *atp6v1e1b* knocked down fins (vivo-MO delivered 2 hpa). In the controls, *mkp3* and *aldh1a2* expression domain was wider at proximal stumps, at both 24 and 48 hpa (Fig. 5 compare F–G; J–K; N–O; R–S). This pattern was somewhat similar to what was observed for the V-ATPase. On the contrary, in V-ATPase knocked down fins, *aldh1a2* and *mkp3* were absent at 24 hpa at either amputation planes, demonstrating that V-ATPase affects the expression of both genes (Fig. 5H–I; P–Q). At 48 hpa, these transcripts' expression was re-established, likely as a consequence of the vivo-MO transient effect (Fig. 5L–M; T–U). Expression of *wnt10a* remained unchanged in control and V-ATPase knocked down fins for all time points, in proximal and distal stumps alike (File S6). These results show that V-ATPase is required for the normal expression of at least *aldh1a2* and *mkp3*, establishing a molecular link between V-ATPase and two important signalling pathways that control cell proliferation, Fgf and RA.

### V-ATPase inhibition affects fin innervation

**Figure 6:**
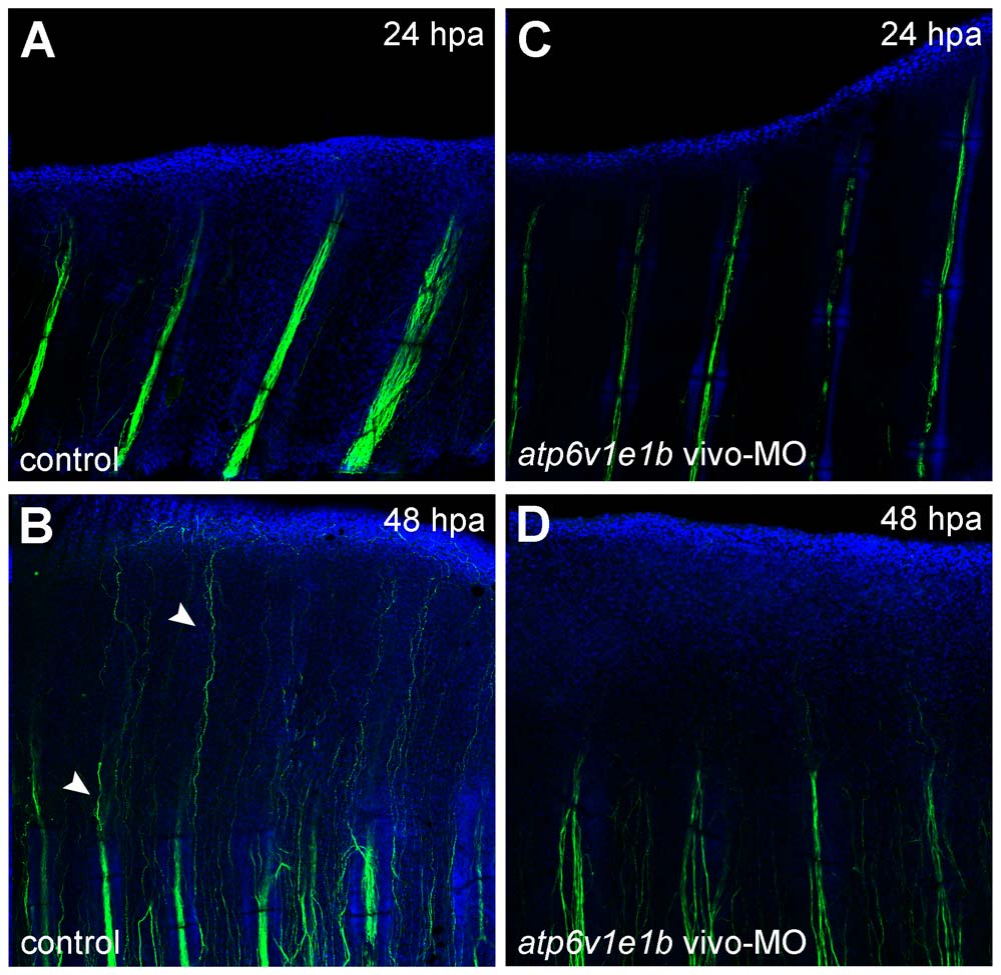
V-ATPase inhibition affects fin innervation. Immunohistochemical detection of acetylated α-tubulin (axons marker) under control regeneration conditions (A, B) and after *atp6v1e1b* knockdown, 2 h after proximal amputation (C, D). (A,C) 24 hpa, (B, D) 48 hpa. Arrow heads: innervation of regenerating tissue. hpa: hours post amputation. For each panel, n = 3.

Proper tissue innervation is another important factor for normal fin regeneration [39]. As such, it was decided to test if any relation to the H^+^ pump existed. For that, *atp6v1e1b* knocked down fins (vivo-MO delivered 2 hpa) were immunostained for the axonal marker acetylated α-tubulin. 24 hours after the proximal amputation of non-treated controls, nerve axons extended mainly along intra-ray regions, forming bundles parallel to the proximal-distal axis of the fin. Actually, these bundles almost reached the amputation plane, after an initial retraction from the stump that typically occurs upon amputation (Fig. 6A). At 48 hpa, the control stumps were innervated by axons sproutings (Fig. 6B). After V-ATPase knockdown, fins were less innervated at 24 hpa and axons remained distant from the amputation plane (Fig. 6C). Later, at 48 hpa, axons were beginning to invade the regenerating tissue in proximal stumps (Fig. 6D). Similar results were found in distal regenerates (File S7). These results show that V-ATPase is required for normal innervation of the regenerating fin.

## Discussion

### V-ATPase activity contributes to a regeneration-specific H^+^ efflux that depends on the amputation position along the proximal-distal axis

Our data showed that H^+^ efflux is specifically established during appendage regeneration in an adult vertebrate. The efflux was first detected several hours upon amputation, and it was present for at least 5 days after wound closure. Previous reports in vertebrates including humans have showed that regeneration is accompanied by an endogenous electric current (EC) for several days after wound healing [13] [14] [40], and this EC seems to be controlled by the activity of specific ion transporters [14] [17]. Since the wound is already closed when an efflux is detected, we propose that H^+^ efflux contributes to the regeneration-specific endogenous EC, probably together with sodium flux, which is a crucial component of regeneration-specific EC, and other ions such as potassium and chloride [13] [14].

The molecular source of regeneration-specific ion fluxes has only recently begun to be unveiled: the H^+^ pump V-ATPase and the voltage-gated sodium channel NaV1.2 are essential for the regeneration of *Xenopus* larvae tail [18] [22], and the H^+^,K^+^-ATPase is required for planarian head regeneration [19]. Nevertheless, evidence in other models for conservation of these mechanisms, especially in adult vertebrate models, is still lacking. In this work, we have showed for the first time that the V-ATPase is upregulated in the regenerating tissue of the adult zebrafish caudal fin. We took advantage of the fact that different manipulations (eg. injection of drugs or morpholinos, amputation planes) can be held on separate parts of one single fin to provide an extra level of control to our experiments that could not be examined in other models such as the *Xenopus* larval tail [18]. We demonstrate that the onset and magnitude of V-ATPase expression varied with the amputation plane along the PD axis and H^+^ efflux followed a similar pattern. Besides, inhibition of this H^+^ pump after either proximal or distal amputation decreased H^+^ efflux. Thus, V-ATPase contributes to the regeneration-specific H^+^ efflux in the adult zebrafish caudal fin.

In the *atp6v1e1b* knocked down fins, the H^+^ efflux decreased at 24 hpa but increased from 48 hpa onwards compared to the control. This phenotype reversal probably reflects the decreasing inhibitory effect of the MO with time [41]. Two interesting facts were that the efflux increase at 48 and 72 hpa closely resembled the control efflux level at 24 and 48 hpa respectively, and for each time point, efflux intensity was always smaller in distal stumps than in proximal ones. Taken together, our data shows that during regeneration V-ATPase activity and the consequent H^+^ efflux are tightly controlled at the genetic level in order to reach a specific intensity that depends on the amputation position. These observations suggest that the V-ATPase has an active role in regeneration and is involved in the formation of position-dependent cues.

### V-ATPase is required for adequate nerve supply and blastema cell proliferation and correlates with RA and Fgf signalling

V-ATPase is ubiquitously expressed in eukaryotic cells and is not only required for several housekeeping functions that depend on pH or membrane potential. Additionally, it is strongly upregulated in particular cell types where it is necessary for specific processes such as bone formation/resorption and renal acidification [42]. In the amputated adult zebrafish caudal fin, V-ATPase was upregulated in the regenerating tissue during blastema formation and early regenerative outgrowth, and its inhibitions impaired regeneration. Therefore, V-ATPase seems to be required for the regenerative process in some role other than housekeeping functions.

In the *Xenopus* tadpole tail, the V-ATPase-mediated H^+^ extrusion is necessary for cell proliferation in the blastema-like regeneration bud [18]. Here, we show that V-ATPase inhibition decreases regeneration by decreasing proliferation in the mature blastema (48 hpa), but not during early blastema formation (24 hpa). Interestingly, H^+^ efflux, mediated by ion transporters such as sodium/proton exchanger 1 (NHE1) and V-ATPase, is a major contributor for cell migration by promoting extracellular matrix degradation [43] [44]. As the blastema arises from intra-ray cells that proliferate and migrate distally [3] [4], V-ATPase could be required for the migration of blastema-forming cells from the intra-ray mesenchyme into the regenerating region.

An alternative view is that cells migrate to the blastema but become unable to proliferate in the absence of V-ATPase. In fact, V-ATPase has been associated to proliferation in other regeneration models [18] [23]. Fgf signalling is required for cell proliferation in the blastema but not in cell populations below the amputation plane [38], and the same influence on cell proliferation was found when V-ATPase activity was inhibited. Besides, V-ATPase knockdown inhibited the expression of *mkp3*, a target of Fgf signalling, demonstrating that this H^+^ pump interferes with this signalling pathway. V-ATPase was also required for the expression of *aldh1a2*, a key molecule for RA synthesis. It is known that both RA and V-ATPase mediate cell survival by upregulating *bcl2* expression [45] [46], so it would be interesting to investigate if the two molecules act as part of a single, common pathway. Importantly, it was recently demonstrated that RA and Fgf signalling, together with Wnt/β-catenin, regulate each other in a positive reciprocal manner and modulate the overall rate of regeneration [47]. Considering this, V-ATPase seems to be another component of this interconnected molecular net by affecting RA and Fgf signalling in a direct or indirect manner. Additionally, an effect of V-ATPase on other signalling pathways, including Wnt/β-catenin, cannot be excluded. Although *wnt10a* was not affect by the pump's inhibition, it has been showed in other systems that V-ATPase is required for Wnt/β-catenin signalling downstream of Wnt ligand [24].

Inhibition of V-ATPase decreased the amount of axons in the fin and inhibited axonal growth into the regenerating tissue. It is possible that this hindered innervation was a direct result of the decrease of V-ATPase mediated H^+^ efflux since it is known that endogenous electric currents and associated electric fields can control the amount of nerve sprouting and the direction of axonal growth into the regenerating region in other models [47]. Besides, in *Xenopus* froglet limb regeneration, nerve supply is not necessary for blastema formation but is required for blastema outgrowth, by controlling cell proliferation, cell survival and expression of Fgf signalling genes in the blastema [39]. Our results showed a similar effect of V-ATPase on blastema cells. Taken all, V-ATPase effect on blastema outgrowth may be mediated by nerve supply, though independent routes for V-ATPase and nerve supply effects cannot be excluded.

### V-ATPase and its putative role in regeneration events that depend on a blastema with specific proliferative function

Although larval fin fold of *atp6v1e1b* ^hi577atg-/-^ zebrafish regenerated normally in the absence of V-ATPase activity, our results clearly show that V-ATPase is required for regeneration in adult fish appendages. In fact, despite the reported conservation of molecular events during regeneration of adult fins and larval fin fold [5] [28], recent *in vivo* cell tracing experiments have showed that the fin fold blastema does not have a specific function for proliferation, and in that way it is not a classical blastema as observed in the adult system [48]. Thus, regeneration of the adult and larval caudal appendages in zebrafish are distinct processes. On the other hand, some remarkable parallels have been observed between regeneration of the adult zebrafish caudal fin and *Xenopus* tadpole limb and tail buds [49], including conserved molecular pathways and dependence on blastema-restricted cell proliferation. Particularly, our work and others [18] demonstrate that, in both models, V-ATPase is required for adequate nerve supply and cell proliferation in the blastema. Taken all, we propose that the V-ATPase has a conserved role in regeneration events that depend on a blastema with specific proliferative function.

### V-ATPase: a novel component of the positional memory transduction system

One important property of the blastema is positional memory, which instructs both the amount and the rate of regeneration so that the missing structures are replaced in the correct 3D pattern and the process is completed simultaneously, regardless the level of amputation along the PD axis [38] [50]. Position-dependent regeneration rate is regulated by the level of expression of several molecules during regeneration [50]. In zebrafish, those include Fgf signalling and *msxb*, which have enhanced proximal expression compared to distally amputated fins [37] [38]. RA is also a major instructor of positional information in several vertebrates, but its role in zebrafish appendage regeneration has proven difficult to assess [51]. We showed that *aldh1a2* has stronger proximal expression compared to distal stumps, adding new evidence that agree with a role for RA in positional memory in zebrafish. Moreover, our results showed that V-ATPase and H^+^ efflux follow a position-dependent pattern with increased proximal expression, while other regeneration markers, such as *wnt10a*, maintain a similar expression regardless the amputation level. Besides, V-ATPase knockdown decreased proliferation in the blastema and inhibited *aldh1a2* and *mkp3* expression. Altogether, these data agree with a role for the V-ATPase in position-dependent regeneration rate, by affecting blastema proliferation through the modulation of at least two essential signalling pathways, Fgf and RA.

To further investigate the role of V-ATPase in position-dependent regeneration rate, we knocked down *atp6v1e1b* at different time points after proximal and distal amputation. The most dramatic regeneration reduction was obtained when the gene knockdown approximated the different onset of H^+^ efflux at proximal and distal positions, around 3 and 12 hpa, respectively. In addition, the decrease in the regenerate area was more pronounced proximally, demonstrating that regions of higher regeneration rate have stronger dependence on V-ATPase activity. This suggests that H^+^ efflux triggers some important mechanism that needs to be activated earlier in the highly proliferating proximal wounds.

Overall, our results suggest that V-ATPase H^+^ pumping activity is part of the signals that translate position into adequate cell behaviour, including position-dependent proliferation rate. In this way, V-ATPase has a role in positional memory system. We propose that some unknown position-instructor signal upstream of V-ATPase sets the level of expression for this H^+^ pump according to the level of amputation along the PD axis. Then, localized V-ATPase H^+^ pumping activity in the blastema generates pH and/or voltage domains within the regenerating tissue, that will act, directly or indirectly (for example, via inhibition of nerve supply), as positive regulators of RA and Fgf signalling, ultimately affecting cell proliferation in the blastema. As V-ATPase gene expression is activated earlier and more strongly in proximal stumps than in distal ones, pH/voltage domains would be more intense and maintained for a greater period of time after proximal amputation. Consequently, V-ATPase activity would exert a stronger influence on gene expression in proximal stumps than in distal ones, including Retinoic Acid and Fgf signalling, ultimately setting a higher regeneration rate in proximal amputated fins.

In 2007, a study from Adams et al. [18] demonstrated that the V-ATPase is necessary for *Xenopus* tadpole tail regeneration. In the present study, our findings further support that the V-ATPase is important to adult vertebrate regeneration. Particularly, our results indicate that the V-ATPase H^+^ pumping activity orchestrates with major molecular signalling pathways to control position-dependent regeneration rate. Understanding of this and other ion-driven mechanisms underlying adult regeneration may open way for new therapeutic strategies, both in regenerative and developmental medicine and in cancer therapy.

## Supporting Information

Table S1 **Ion-specific electrodes optimized to measure ion-specific flux in the adult zebrafish fin, using SIET.**
(XLS)

Table S2 **Ion-specific recording medium for SIET- based ion-specific flux measurement in the adult zebrafish fin.**
(XLS)

Table S3 **Sequence-specific primers** for cloning of gene-specific fragments for *atp6v1e1b*, *atp6v1a*, *wnt10b*, *mkp3* and *aldh1a2* by PCR amplification from zebrafish cDNA.
(XLS)

File S1 **Ion-specific fluxes during caudal fin regeneration.** SIET-mediated detection of potassium (K^+^, Figure **A**), sodium (Na^+^, Figure **B**), calcium (Ca^2+^, Figure **C**) and chloride (Cl^-^, Figure **D**) flux patterns at the ray and inter-ray regions, during regeneration. For the four ion-species, there was a high efflux upon amputation (0.08 hpa) that rapidly decreased as the wound closed (6 hpa), becoming similar to intact fins (0 hpa) from 24 hpa.
(TIF)

File S2 **V-ATPase subunits localization in chloride cells, intact and regenerating fins.** Whole mount *in situ* hybridization for *atp6v1e1b* (Figure **A**) and *atp6v1a* (Figure **B**) in intact fins. Cross section of the whole mount *in situ* hybridization for *atp6v1a* at 24 hours post amputation (hpa) where expression can be observed in the blastema, distal to the bone (Figure **C**, arrowheads). Atp6v1a in the intact caudal fin is present mainly in the epidermis, in a scattered pattern (Figure **D**). Whole mount *in situ* hybridization (Figure **E**) and immunostaining (Figure **F**) for atp6v1a subunit in the chloride cells of the zebrafish embryo.
(TIF)

File S3 **Delivery of control and** ***atp6v1e1b*** **fluorescein-tagged morpholinos.** Merged fluorescent and bright field image showing the incorporation of both control and *atp6v1e1b* fluorescein-tagged morpholinos into opposite regions of a regenerating fin, 24 h after delivery.
(TIF)

File S4 **V-ATPase inhibition does not affect apoptosis during regeneration.** *atp6v1e1b* knockdown didn't affect cell proliferation by 24 hpa compared to the control (compare Figures **A-A’** with **B-B’**).
(TIF)

File S5 **V-ATPase is required for cell proliferation in the mature blastema after amputation at the distal plane.** *atp6v1e1b* knockdown didn't affect cell proliferation by 24 hpa (compare Figures **A** and **C**; **E**). However, at 48 hpa there were significantly less blastema cells positive for H3P than in the control (compare Figures **B** and **D**; **E**).
(TIF)

File S6 **Expression of** ***wnt10a*** **is not affected by V-ATPase knockdown.** *In situ* hybridization for *wnt10a* showed a stronger/wider expression in proximal stumps than in distal ones, both at 24 and 48 hpa under normal regeneration conditions (compare Figures **A** and **B**, Figures **E** and **F**). This pattern remained unchanged upon *atp6v1e1b* knockdown (compare Figures **C** and **D**, Figures **G** and **H**).
(TIF)

File S7 **V-ATPase inhibition affects fin innervation after distal amputation.** Fins were immunostained for acetylated α-tubulin under normal regeneration conditions (control) and after *atp6v1e1b* knockdown at 2 hpa. V-ATPase knockdown decreased fin innervation both below the amputation plane and in the regenerating tissue (compare Figures **A**–**B** with **C**–**D**, respectively).
(TIF)
