# Supplementary figures and images for: V-ATPase Proton Pumping Activity Is Required for Adult Zebrafish Appendage Regeneration
Source: PLoS One. 2014 Mar 26;9(3):e92594. doi: 10.1371/journal.pone.0092594 (PMC3966808; doi:10.1371/journal.pone.0092594)

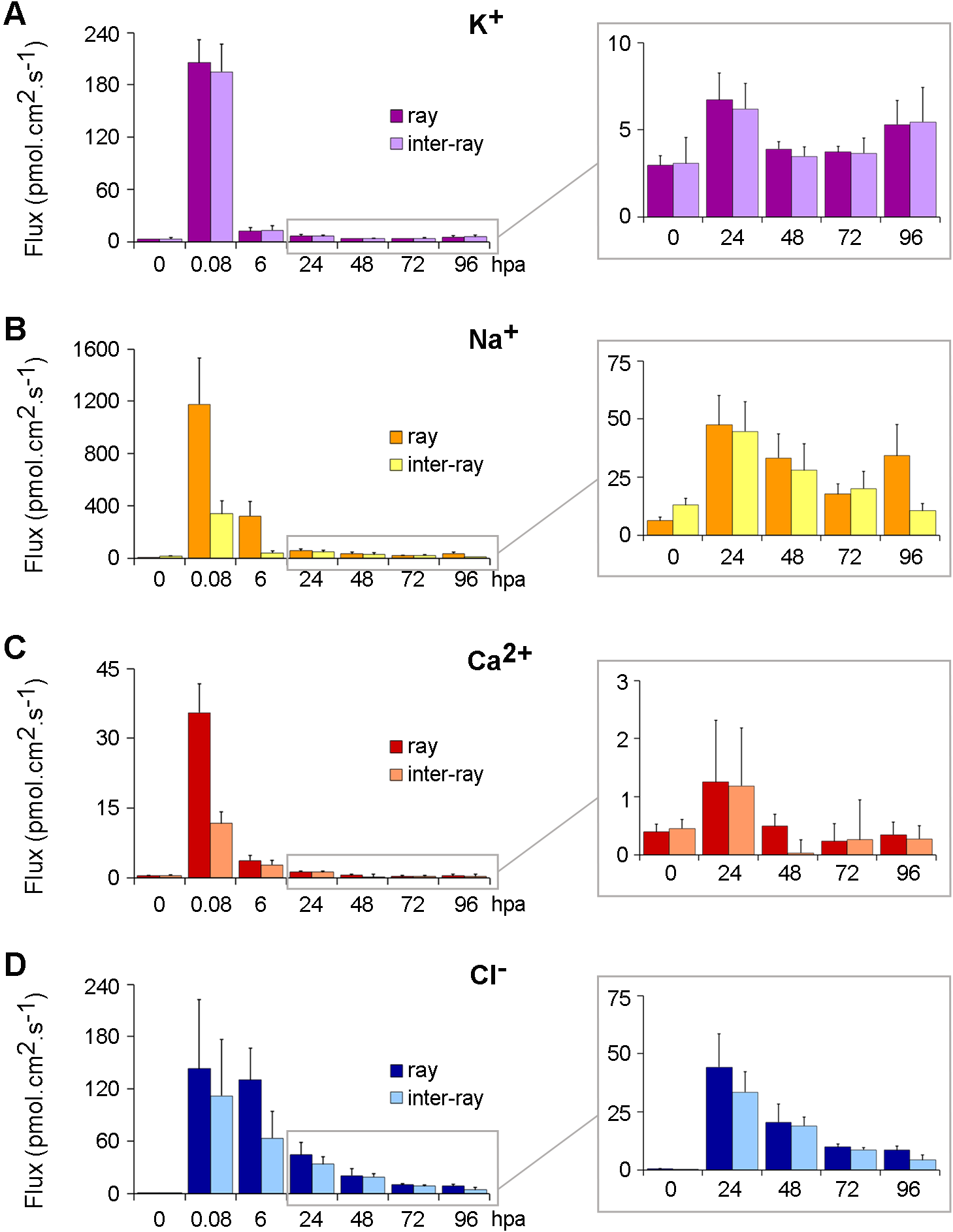

Supplement: File S1 — Ion-specific fluxes during caudal fin regeneration. SIET-mediated detection of potassium (K+, Figure A), sodium (Na+, Figure B), calcium (Ca2+, Figure C) and chloride (Cl-, Figure D) flux patterns at the ray and inter-ray regions, during regeneration. For the four ion-species, there was a high efflux upon amputation (0.08 hpa) that rapidly decreased as the wound closed (6 hpa), becoming similar to intact fins (0 hpa) from 24 hpa. (TIF) [file pone.0092594.s004.tif]

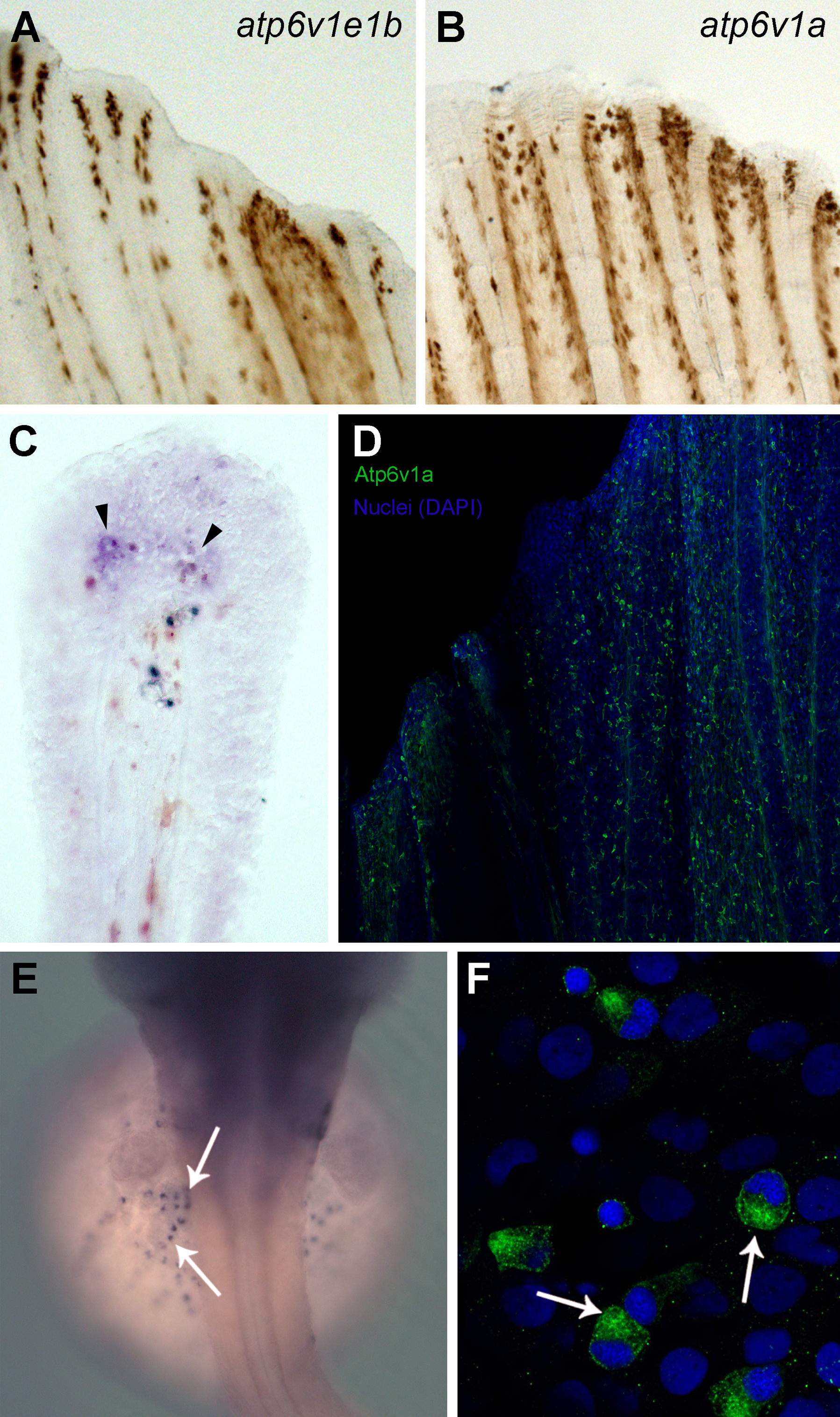

Supplement: File S2 — V-ATPase subunits localization in chloride cells, intact and regenerating fins. Whole mount in situ hybridization for atp6v1e1b (Figure A) and atp6v1a (Figure B) in intact fins. Cross section of the whole mount in situ hybridization for atp6v1a at 24 hours post amputation (hpa) where expression can be observed in the blastema, distal to the bone (Figure C, arrowheads). Atp6v1a in the intact caudal fin is present mainly in the epidermis, in a scattered pattern (Figure D). Whole mount in situ hybridization (Figure E) and immunostaining (Figure F) for atp6v1a subunit in the chloride cells of the zebrafish embryo. (TIF) [file pone.0092594.s005.tif]

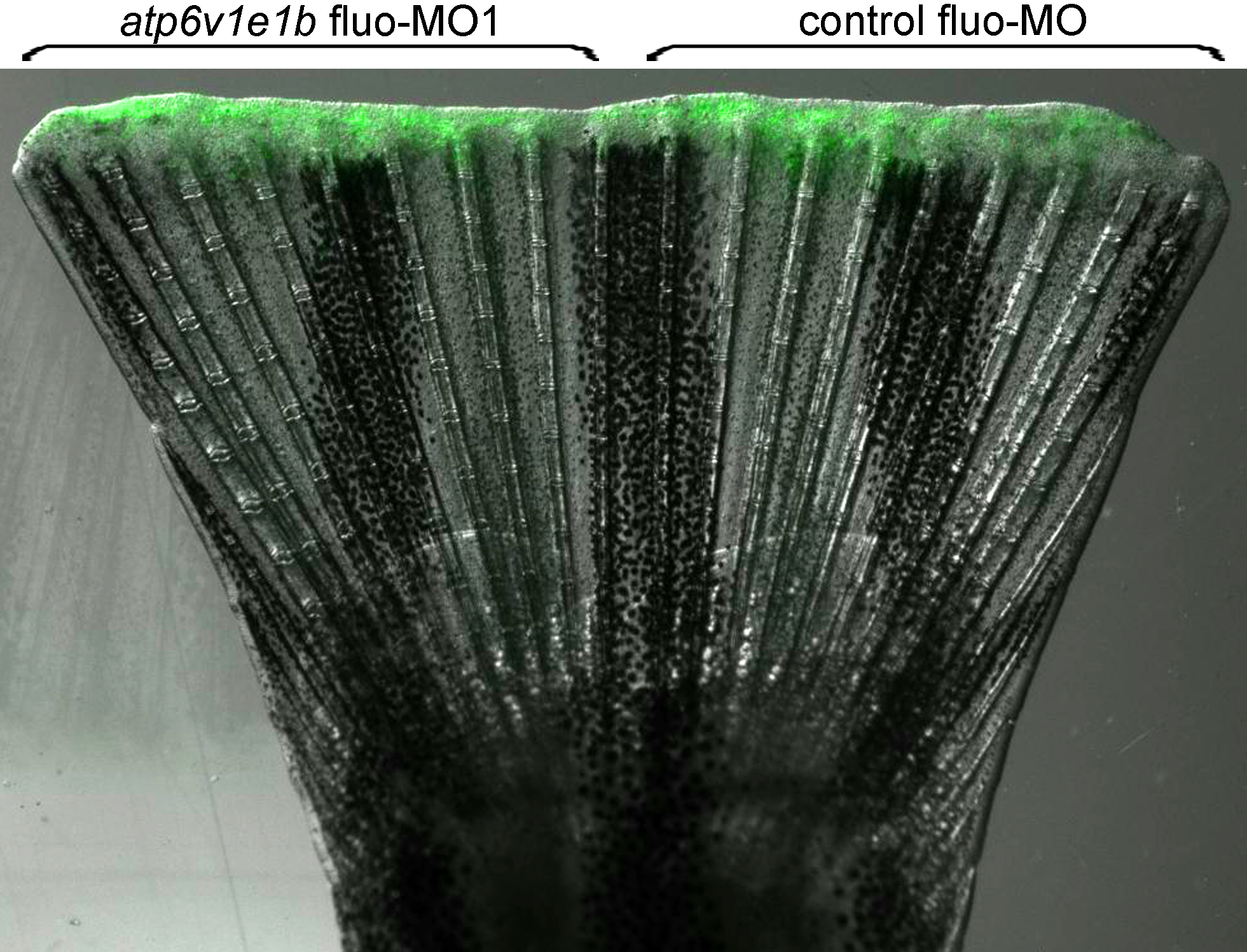

Supplement: File S3 — Delivery of control and atp6v1e1b fluorescein-tagged morpholinos. Merged fluorescent and bright field image showing the incorporation of both control and atp6v1e1b fluorescein-tagged morpholinos into opposite regions of a regenerating fin, 24 h after delivery. (TIF) [file pone.0092594.s006.tif]

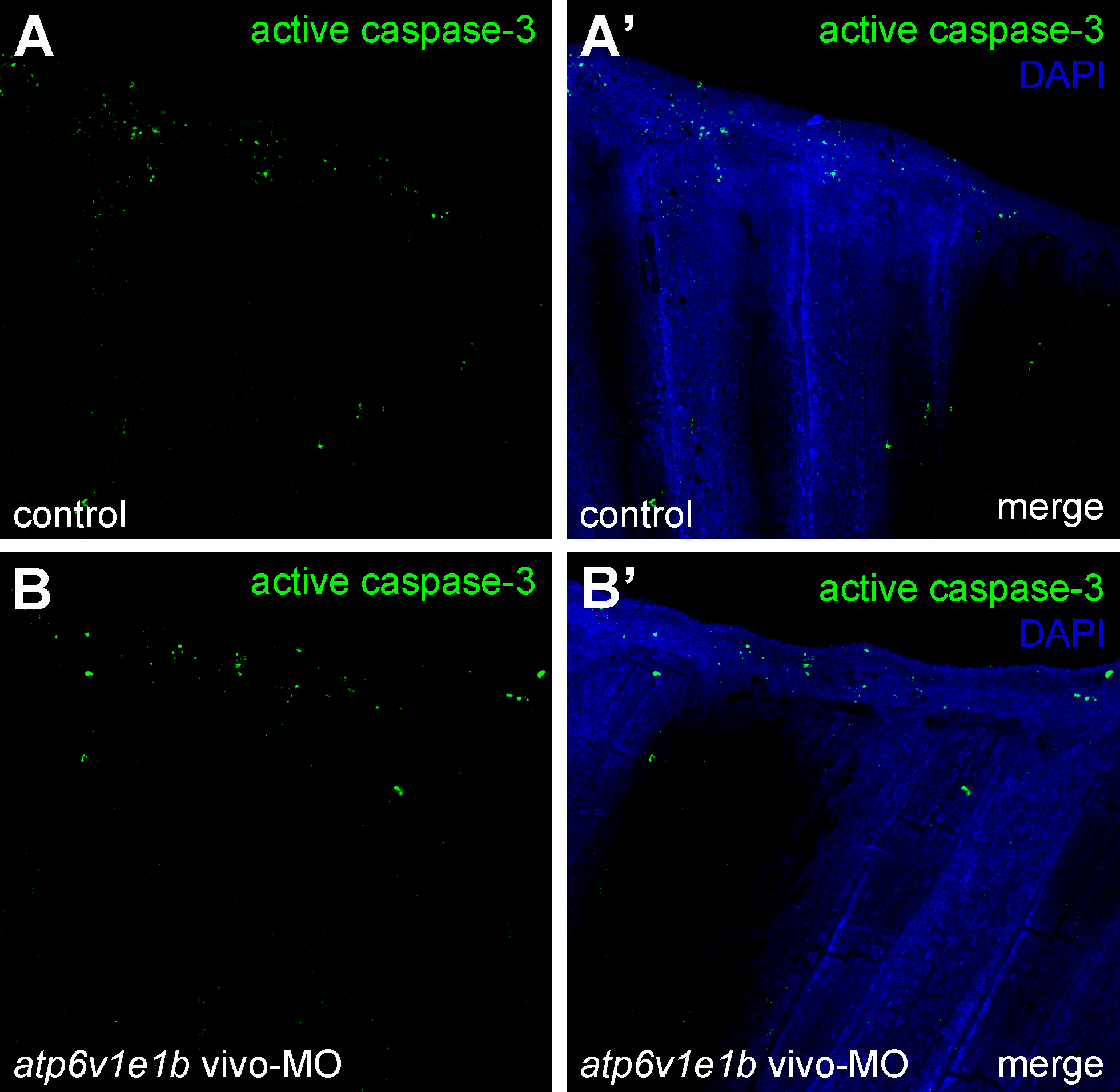

Supplement: File S4 — V-ATPase inhibition does not affect apoptosis during regeneration. atp6v1e1b knockdown didn't affect cell proliferation by 24 hpa compared to the control (compare Figures A-A’ with B-B’). (TIF) [file pone.0092594.s007.tif]

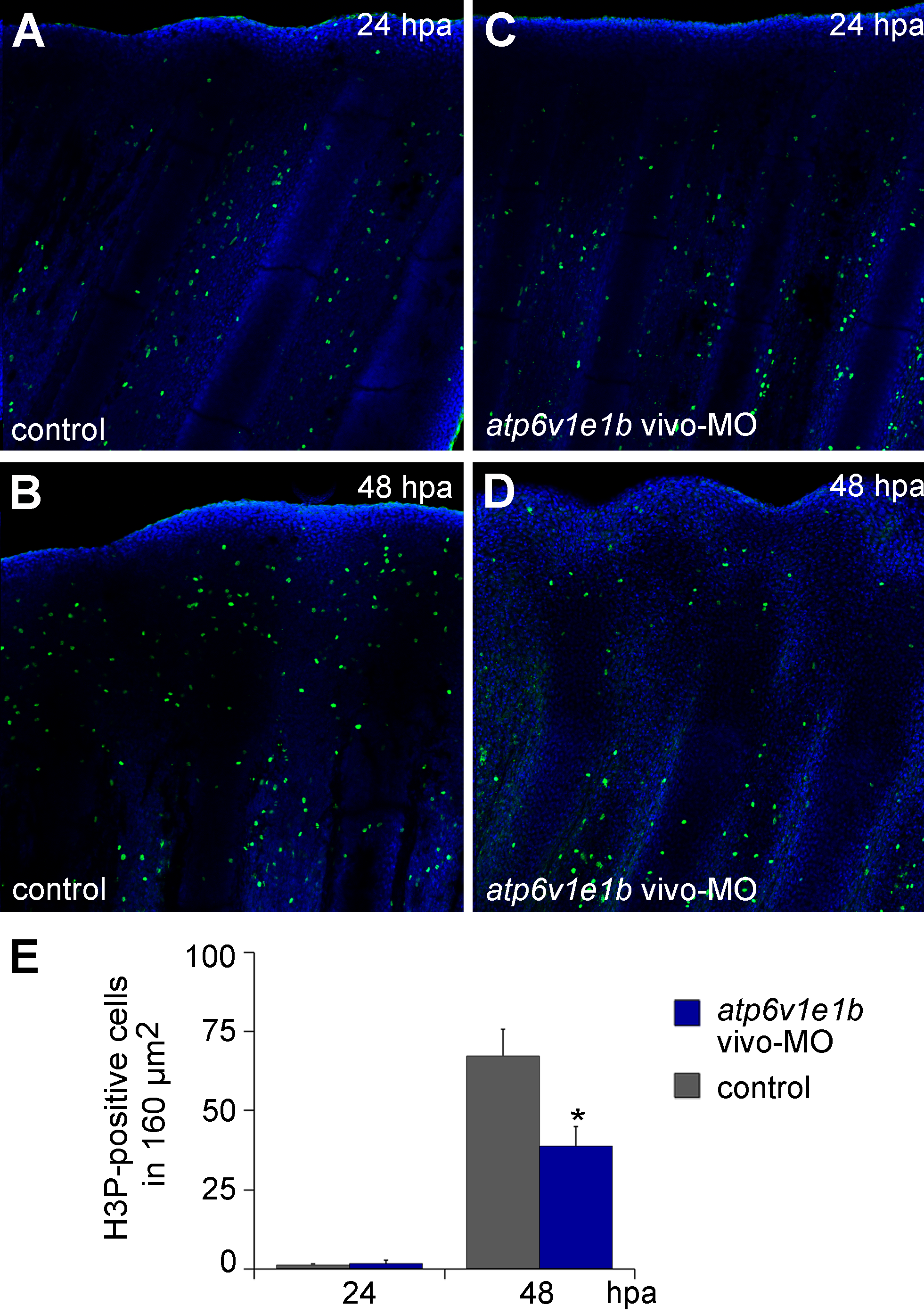

Supplement: File S5 — V-ATPase is required for cell proliferation in the mature blastema after amputation at the distal plane. atp6v1e1b knockdown didn't affect cell proliferation by 24 hpa (compare Figures A and C; E). However, at 48 hpa there were significantly less blastema cells positive for H3P than in the control (compare Figures B and D; E). (TIF) [file pone.0092594.s008.tif]

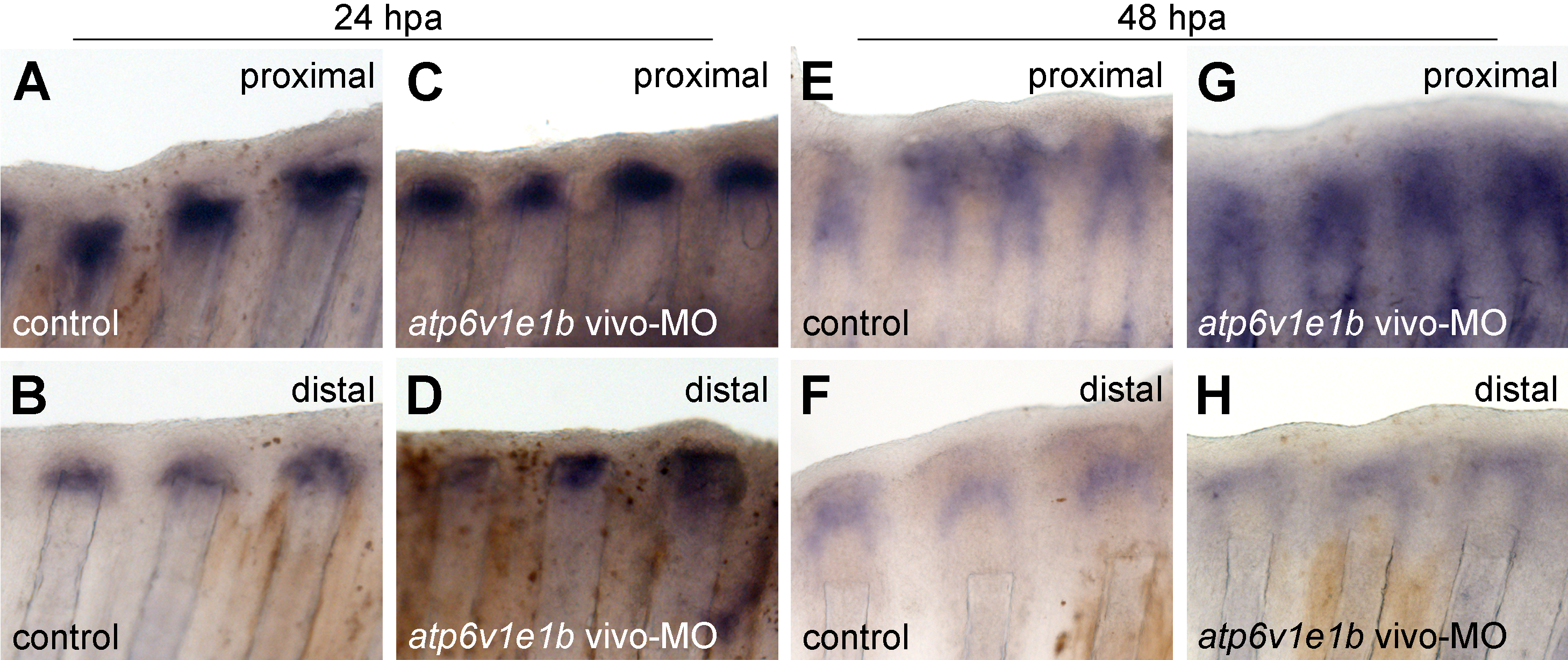

Supplement: File S6 — Expression of wnt10a is not affected by V-ATPase knockdown. In situ hybridization for wnt10a showed a stronger/wider expression in proximal stumps than in distal ones, both at 24 and 48 hpa under normal regeneration conditions (compare Figures A and B, Figures E and F). This pattern remained unchanged upon atp6v1e1b knockdown (compare Figures C and D, Figures G and H). (TIF) [file pone.0092594.s009.tif]

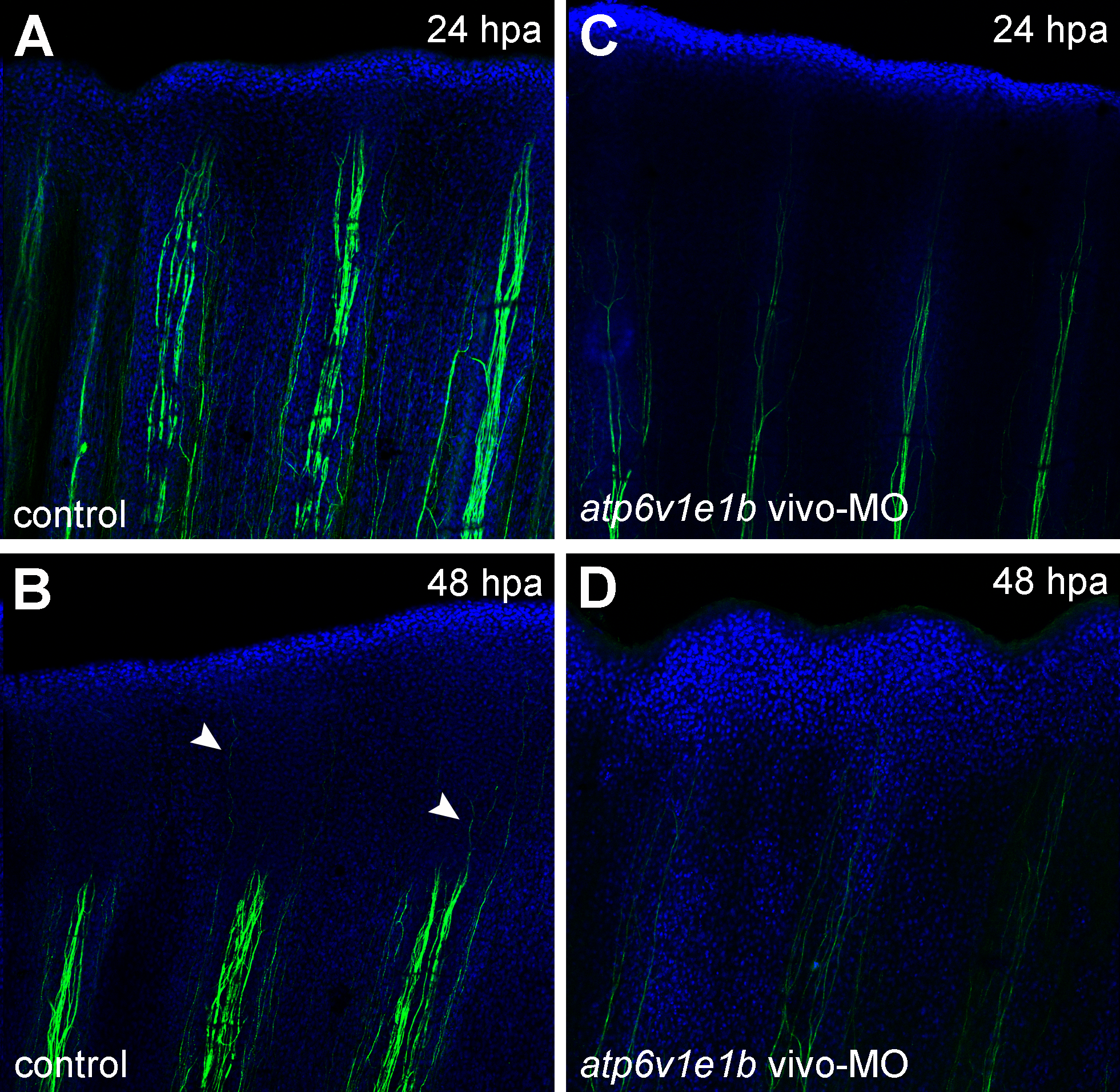

Supplement: File S7 — V-ATPase inhibition affects fin innervation after distal amputation. Fins were immunostained for acetylated α-tubulin under normal regeneration conditions (control) and after atp6v1e1b knockdown at 2 hpa. V-ATPase knockdown decreased fin innervation both below the amputation plane and in the regenerating tissue (compare Figures A–B with C–D, respectively). (TIF) [file pone.0092594.s010.tif]
